# Colour-Value Based Method for Polydopamine Coating-Stability Characterization on Polyethersulfone Membranes

**DOI:** 10.3390/membranes7040070

**Published:** 2017-12-16

**Authors:** Thomas Bucher, Juliana I. Clodt, Andrej Grabowski, Martin Hein, Volkan Filiz

**Affiliations:** 1 Institute of Polymer Research, Helmholtz-Zentrum Geesthacht, Max-Planck-Str.1, 21502 Geesthacht, Germany; thomas.bucher@hzg.de (T.B.); Juliana.Clodt@hzg.de (J.I.C.); 2 MAHLE International GmbH, Pragstr. 26-46, 70376 Stuttgart, Germany; andrej.grabowski@prof-ed.eu (A.G.); martin.hein@mahle.com (M.H.)

**Keywords:** microfiltration, ultrafiltration, polydopamine, coating, stability, temperature dependent fluxes, polyethersulfone membranes

## Abstract

Porous polyethersulfone membranes as used in oenology were investigated in order to evaluate temperature-dependent permeances in a temperature range from 10 to 35 °C. A temperature correction factor was determined for this type of membrane to get accurate and comparable results for further developments. Moreover, the membranes were modified with a bio-inspired polydopamine coating in order to reduce fouling. The performance of the membranes could be increased with respect to permeance and flux recovery under cross-flow conditions. In order to test the applicability and stability of the coating layer, they were treated with basic and acidic cleaning agents as used in industry for fouled membranes. The chemical stability of the coating layer was studied under basic and acidic conditions, by systematic observation of the colour change of the coated membranes over treatment time.

## 1. Introduction

**Figure 1:**
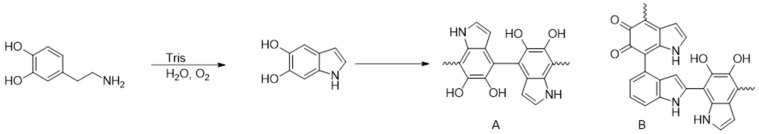
Possible mechanism of polydopamine formation and possible structures (**A**,**B**) of polydopamine [32].

Porous membranes as used in ultrafiltration (UF) or microfiltration (MF) work energy efficiently under low pressures. Their broad ranges of applications vary from water treatment [1,2,3], sterilization, and biomolecule retention [4,5], to whey, juice, beer and wine filtration [4,6,7]. Membranes implemented in the latter field are generally made from polymers such as cellulose acetate, polypropylene, polysulfone, polyvinylchloride and polyethersulfone [6]. One major problem in membrane technology is the difficulty to control fouling, leading to increasing costs due to production interruptions, since the membranes have to be cleaned after defined periods of time. Consequently, one special interest of membrane scientists as well as the membrane industry is the reduction of fouling. On the one hand, novel cleaning methods are under investigation; on the other hand, different membrane materials or membrane modification methods [8,9,10,11] to prevent or reduce fouling play important roles within this research topic [12,13,14]. In the field of surface modification, the mussel-inspired polydopamine coating has attracted scientists in the last couple of years [15,16,17,18,19,20,21,22,23,24,25,26,27]. Based on an oxidative polymerization with oxygen or oxidant induced [28] polymerization of dopamine hydrochloride to polydopamine, this coating can be carried out under slightly basic conditions (see Figure 1) [29,30]. Nevertheless, the structure of polydopamine is not completely understood and still under discussion (suggested structures shown in Figure 1) [31,32,33].

Polydopamine offers large potential in application as coating or as an interlayer for further reactions like Michael addition. Recently, it was hypothesised that biofunctionalized conductive polymers based on polydopamine enable even metal-free efficient CO_2_ electroreduction [34]. In the area of porous membranes, polydopamine coatings were under investigation to increase hydrophilicity [35], or for providing the possibility for further modifications to advance separation performance or antifouling behavior [36].

In this work, we compared flat sheet polyethersulfone membranes before and after polydopamine coating with different coating times. A temperature correction factor to normalize temperature fluctuations was determined for the polyethersulfone membranes, adjusted to the temperature range 18–25 °C in order to obtain accurate and comparable results. After investigation of the mean water flux, the effect of the coating on the pure water permeance in dead-end-mode was investigated. Additionally, the modification with different polydopamine coating times was studied with contact angle measurements. Furthermore, the permeance and the fouling behaviour of the modified and pristine membranes were studied under cross flow conditions. Stability tests of the polydopamine coating against basic or acidic cleaning agents as used in industry were carried out. For this purpose we investigated a method to determine the degree of degradation of the polydopamine layer by examination of the colour value change against the cleaning time.

## 2. Results and Discussion

In this work, we first studied the temperature dependent permeances of polyethersulfone (PES) membranes in order to get accurate data for the permeances generally measured at room-temperatures that may differ from 18 to 25 degrees. Therefore, a correction factor had to be found. The PES membranes were coated with polydopamine, their performance were examined and the stability of the coating was tested against chemical cleaning as used in industry. In order to determine the fouling on coated and uncoated membranes, permeance measurements similar to cross flow including backwash cycles were performed and discussed.

### 2.1. Temperature Dependent Water Permeances of PES Membranes

**Figure 2:**
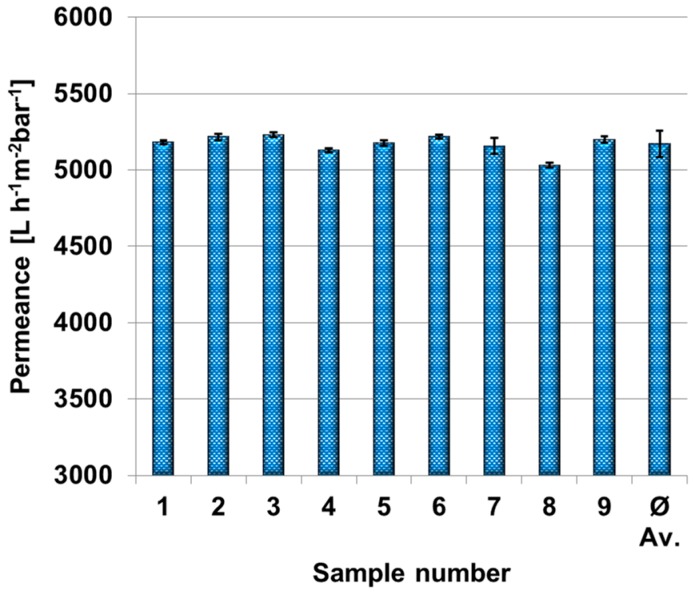
Permeance of nine polyethersulfone membrane samples and average value in dead-end mode. Transmembrane pressure was set to one bar. Each permeance and standard deviation were calculated out of 10 different measurement points after 10 min.

In order to determine the homogeneity of the PES membranes and the reproducibility of the permeances for different coating times, the water permeance was measured for nine PES membrane samples selected randomly on the membrane sheet. Therefore, each membrane sample was measured over 20 min. Data points were collected every 60 s and the average value of the last 10 data points including error bars are shown in Figure 2. The average permeance of these nine membrane sheets is 5221 L·h^−1^·m^−2^·bar^−1^ with a standard deviation of 3.4%. We concluded from these measurements that the polyethersulfone membrane load is uniform enough for our further purpose.

**Figure 3:**
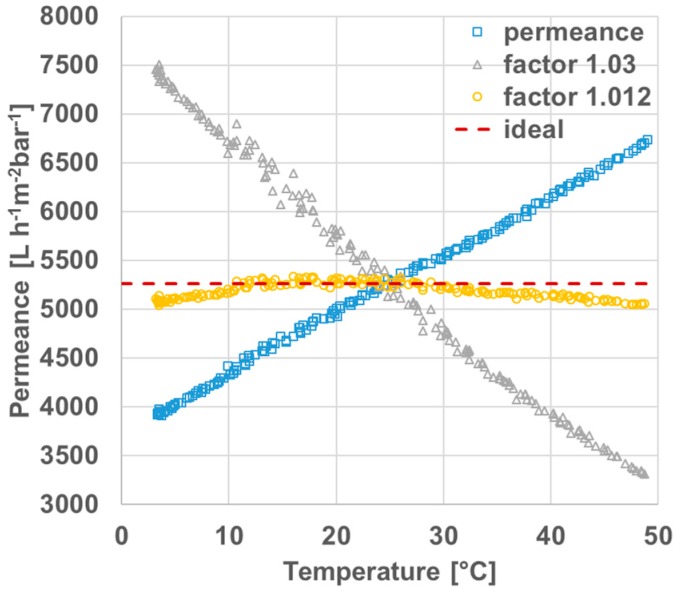
Temperature-dependent permeances of polyethersulfone membranes: original measured permeance (squares), including correction factor of 1.03 as suggested in the literature [37] (triangles), including correction factor of 1.012 determined in this work (circles) and ideal graph (dashes).

The permeances of polyethersulfone membranes were measured between 3 and 50 °C in order to determine a temperature correction factor and get accurate and comparable data for the permeances at room temperature that may range from 18 to 25 °C. Figure 3 depicts amongst others the uncorrected permeances (yellow squares) and the corrected permeances (blue triangles) using the temperature correction factor of 1.03 as suggested from the literature [37]. Uncorrected permeances increase with the temperature as estimated. The slope of the temperature corrected data (TCF = 1.03) is negative, even though an approximation to the ideal linear curve, red dashes, was expected. Therefore, we determined the temperature correction factor especially for these types of polyethersulfone membranes as 1.012 and the resulting data is shown as orange circles in Figure 3. The curve is in good agreement with ideal data between 10 and 30 °C and reasonable from 3 to 10 °C and 30 to 48 °C. This will cover typical room-temperatures found in different laboratories that may be not air-conditioned and consequently are subject of seasonal influences.

### 2.2. Modification of the Membranes and Proof of Successful Coating

**Figure 4:**
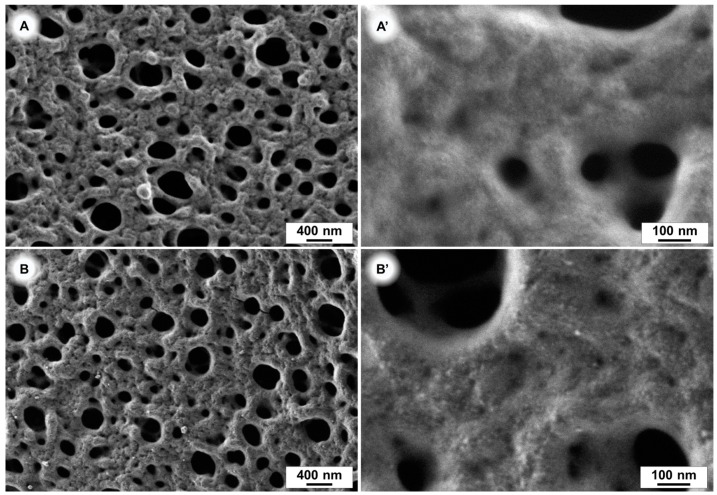
SEM images of polyethersulfone (PES) membranes. Uncoated (**A**,**A**’) and after 240 min polydopamine coating (**B**,**B**’).

In order to prove a successful coating of our membranes scanning electron microscope (SEM) images, atomic force microscopy (AFM), colour-value determination on photos, contact angle measurements and permeance measurements were carried out. SEM images of a pure and a modified PES membrane, after 240 min of polydopamine coating time, are depicted in Figure 4A,B. After this coating time we did not observe a significant difference in the pore size of the membranes with SEM, indicating that the polydopamine layer is very thin. The pore size reduction due to polydopamine coating is therefore negligible. The successful coating of 240 min coated samples is illustrated in Figure 4B’, which shows a higher surface roughness caused by the polydopamine (PDA) layer compared to the pristine membrane (Figure 4A’).

**Figure 5:**
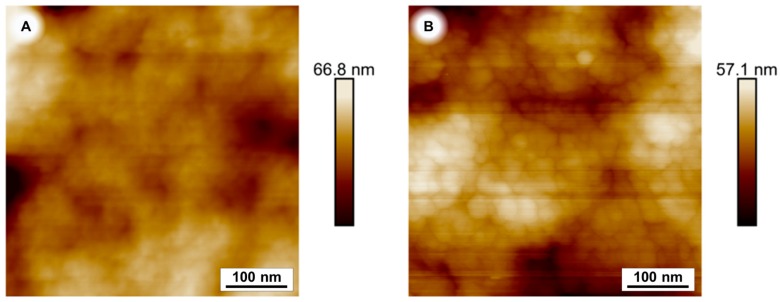
Atomic force microscopy (AFM) image of PES membranes. Uncoated (**A**) and after 240 min polydopamine coating (**B**).

Atomic force microscopy images of a coated and an uncoated sample emphasise the change of the surface morphology of PES membranes after a 240 min PDA coating time. (Figure 5).

**Figure 6:**
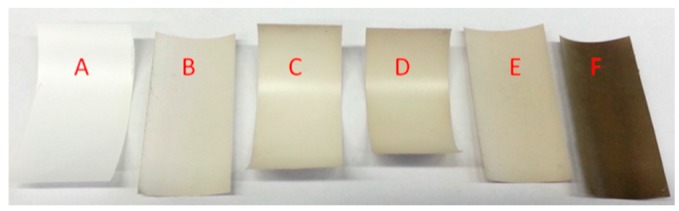
Time dependent polydopamine coating; (**A**) pure membrane, (**B**) 120 min (**C**) 180 min, (**D**) 240 min, (**E**) 300 min, (**F**) 69 h of coating.

The darker colour of membranes with increasing coating time is an indication of the polydopamine thickness on a substrate due to the strong broad band UV-visible absorption [38]. This can be simply observed by photography. PES membranes were coated with polydopamine for different times. Figure 6 depicts the resulting membranes dependent on the coating time. We concluded a 240-min dopamine coating to be at least sufficient for our purpose, since we were looking for the lowest possible but still uniform polydopamine coating.

#### 2.2.1. Thermogravimetric Analysis (TGA)

**Figure 7:**
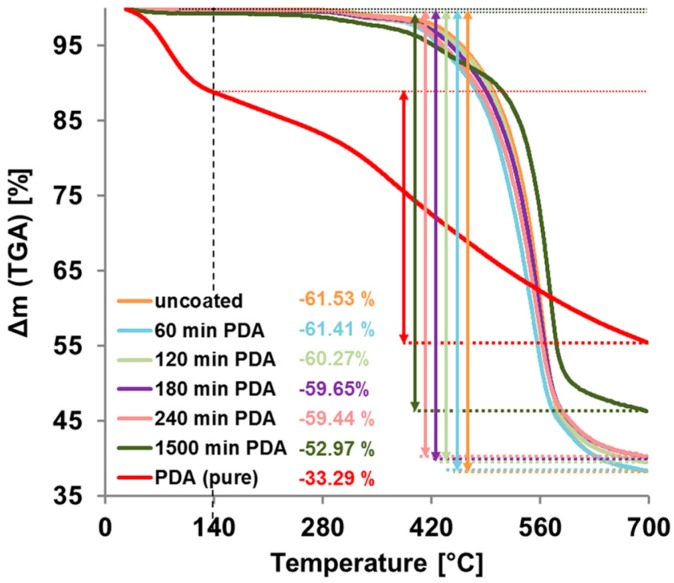
Thermogravimetric Analysis (TGA) measurements of uncoated and polydopamine (PDA)-coated PES membranes and of pure PDA. The mass loss between 140 °C and 700 °C is marked with coloured arrows and the value is given for each sample.

The results of the thermogravimetric analysis are shown in Figure 7. Free PDA particles filtered out of a reaction solution without sample, showed stronger mass loss between 27 and 140 °C probably due to water or crystal water. As expected, the mass loss of pristine and coated membrane samples in this range is very small. Comparing the change of mass between 140 and 700 °C, we found a mass loss of −61.5% for the pristine PES membrane and −33.3% for free PDA-particles. The mass loss of coated PES membrane samples are in between; the longer the coating time, the closer they come to pure PDA. These results verify the expected growth of the PDA layer with increasing reaction time.

#### 2.2.2. Gravimetric Analysis

To investigate the mass deposition of polydopamine on PES membrane samples, we measured the mass difference gravimetrically before and after coating with samples of two by four centimeters in size. While the mass change of the sample after 60 min coating time was only 0.03 mg, which is in the range of the measuring error of our scale (±0.02 mg) (equals 37 mg/m^2^ (±25)), this error got smaller when more PDA was deposited on the samples, for longer coating times therefore.

The mass change itself is a proof of PDA deposition on membrane samples, though there is no information about the coating thickness, for a 240-min PDA coated.

#### 2.2.3. Contact-Angle Measurement

**Figure 8:**
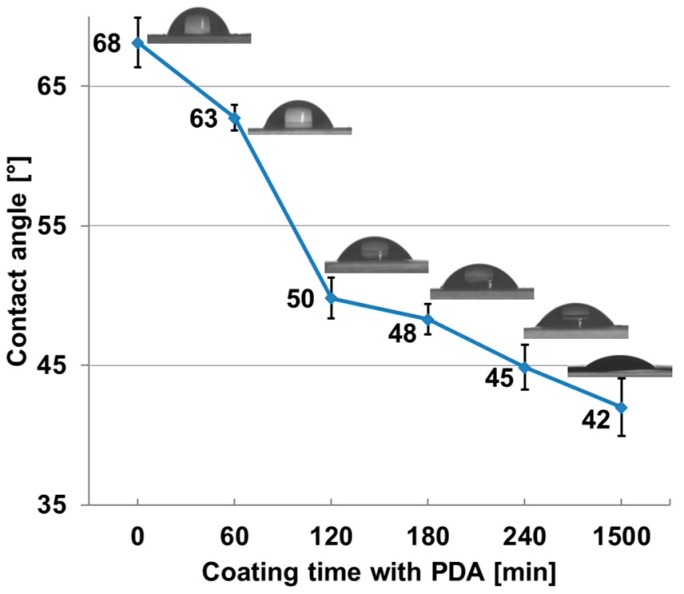
Contact angle and snapshots of a pure PES membrane and PES membranes after different coating time with polydopamine.

Contact angles were measured for the pure membrane and several polydopamine coating times and the results are depicted in Figure 8. As expected, the contact angle measurements indicate clearly that the surfaces of the membranes are more hydrophilic after modification with polydopamine. The contact angles decrease with increasing coating time most intense between 60 min and 120 min of coating time.

The permeance increases of polyethersulfone membranes after different polydopamine coatings times from 60 to 240 min is shown in Table 1. The ultrathin coating does not significantly reduce the pore size, but strongly effects the hydrophilicity, which seems to have the bigger influence on the pure water flux. This effect was previously explained by opening of inaccessible channels of more hydrophic uncoated membranes or by a slipping effect of water on the more hydrophilic material in small channels, where the rise of hydrophilicity is caused by the hydroxyl groups of the PDA coating [36,39,40]. However, the permeance increases with the coating time up to 10.4% for 240 min of coating. Further coating up to 1500 min showed a decline of this effect and the permeance increase goes down to 0.4%, which is expectable, because the hydrophilicity could not be increased further significantly between 240 min and 1500 min, while the pore size shrunk further and, additionally, after 1500 min, big particles of PDA formed within the reaction solution, that may also partly block some pores.

While the highest increase of permeance with 4.6% takes place after 60 min of polydopamine deposition, the effect slows down with ongoing reaction time, which is 6.6% after 120 min, 9.2% after 180 min and 10.4% after 240 min of coating time, respectively (see Table 2).

### 2.3. Stability of Polydopamine against Chemical Cleaning

**Figure 9:**
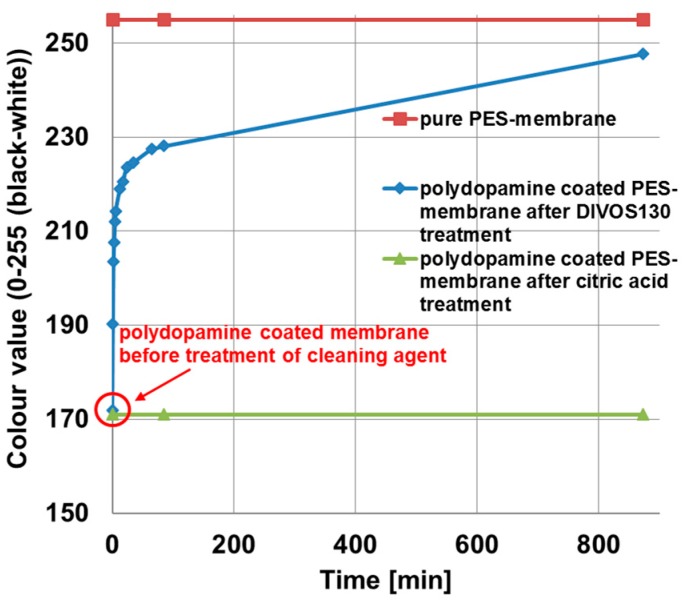
Stability study of PDA-coated PES membranes against chemical membrane cleaning agents: pure polyethersulfone membrane (red squares); after DIVOS130 treatment, containing mainly NaOH and EDTA (blue rhombs); and after two percent citric acid treatment (green tringles). The graph shows the change of the colour value with ongoing cleaning time, the value 0 means a completely black sample and 255 an uncoated polyethersulfone membrane.

Chemical cleaning of membranes as used in industry was carried out under basic or acidic condition in order to remove foulants like natural organic matter from the membrane surface. Therefore, we tested the stability of polydopamine coating on polyethersulfone membranes against DIVOS130 (2 wt %; pH = 12.5), a basic cleaning agent typically used in oenology, and against citric acid (2 wt %; pH = 2.6), as acidic cleaning agent for membranes. Since polydopamine coatings exhibit a typical brownish colour, the colour value change of the coated membrane after deposition to cleaning agents can be examined in order to study the stability of the coating. Before proceeding, we ensured these cleaning agents did not cause any change of the membrane color by treating the pristine PES membrane without any coating under the same conditions. This was expectable due to the chemically inert nature of PES against this class of cleaning agents. Figure 9 depicts the results of the stability test. The pure PES membrane is shown at a colour value of 255 (red squares), the colour value for polydopamine coated membranes before cleaning agent treatment at 172, increases quickly with increasing DIVOS130 treatment time (blue rhombs) at the beginning. The steep slope became flatter after 50 min, whereupon most of the polydopamine coating layer is already removed. An explanation for the varying slope could be the nature of the coating and the membrane surface, respectively. Most of the polydopamine will be bound on the surface or within the bigger open pore structure, because this is the best accessible area during the coating process. Hence it is also easily exposed to the cleaning agent. The smaller pores or small chambers where polydopamine is entrapped and better protected can adhere a bit longer, while the slope decreases after 50 min. In case of citric acid treatment, the colour value stays constant at 172 even after 800 min.

This stability test against chemical cleaning reveals clearly that polydopamine coatings as carried out in his work are not stable in DIVOS130 and consequently are not suitable for application in membrane industry where DIVOS130 or similar basic cleaning agents must be used to clean the membranes. This observation is in good agreement with previous work indicating that the detachment of polydopamine films on various substrates was high for 0.1 M NaOH solution [41].

Under basic conditions, polydopamine shows a negatively charged surface due to the deprotonation of its phenolic hydroxyl groups [42], where the repulsive charges may lead to the fast deatachment.

Other cleaning methods must be applied like acidic cleaning with citric acid indicating a good stability with this treatment.

### 2.4. Fouling Test under Cross-Flow Condition

**Figure 10:**
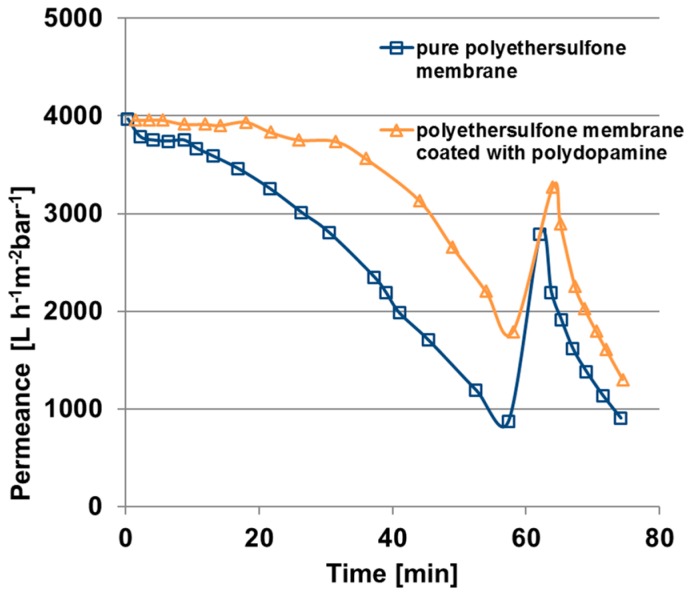
Fouling behaviour under cross-flow condition of a pure polyethersulfone membrane and a polydopamine membrane coated for 240 min, fouling solution: 61 mg/L BSA in water, transmembrane pressure one bar, velocity 0.97 m/s.

In order to estimate the fouling behaviour of the polydopamine coated membranes, cross flow filtrations (velocity 0.97 m/s) of BSA solutions were carried out with coated and uncoated membranes. The results are depicted in Figure 10 and the calculated permeance declines, reversible and irreversible fouling ratios according to Equations (4) and (5) (see Materials and Methods) are listed in Table 3. Within 60 min of BSA filtration the pure polyethersulfone membrane has a permeance decline of 78% (blue squares) whereas the flux of the coated membrane decreases only 55% (yellow triangles).

Flux recovery is increased for the coated membrane and, significantly, the irreversible fouling ratio decreases from 30 to 17% for the polydopamine modified membrane, which plays an important role when it comes to application. However, for both types of membrane the initial permeance could not be achieved after the backwashing cycle of one minute. Consequently, the hydraulic cleaning as carried out in this work is not sufficient enough since BSA is adsorbed insight the pores and the substructure of the membrane. It has to be mentioned that this initial fouling test over 80 min gives only an indication about the fouling behaviour.

## 3. Materials and Methods

### 3.1. Materials

PES membranes were prepared by the phase inversion process from MAHLE International GmbH (Stuttgart, Germany). The membranes were rinsed with iso-propanol before use and stored in water. Bovine serum albumin (BSA), tris(hydroxymethyl)-aminomethan-hydrochloride (Trizma HCl), tris(hydroxymethyl)-aminomethan (Trizma base), dopamine hydrochloride, citric acid monohydrate were purchased from Sigma Aldrich (St. Louis, MO, USA); DIVOS 130 as used for membrane cleaning in oenology is a mixture of sodium hydroxide, EDTA, anionic surfactants, sodium salts, ethylenediaminetetraacetic acid and was purchased from Diversey (Charlotte, NC, USA); iso-propanol, analytical grade, was purchased from Merck KGaA (Darmstadt, Germany). All compounds were used without further purification.

### 3.2. Polydopamine Coating

PES membranes (approx. 2 cm × 4 cm, samples of 2 cm diameter, respectively) were dipped into a solution of dopamine hydrochloride (2 mg/mL) dissolved in 15 mM Tris-buffer (pH 8.5–8.8, ultrapure water). The reaction vessel was placed on a shaker and shacked for a defined time (60 min up to 2 days). The membranes were rinsed three times with 50 mL of ultrapure water for 30 min and stored in water. In order to measure SEM images the membranes were dried at 60 °C under reduced pressure for three days.

### 3.3. Thermogravimetrical Analisis (TGA)

Thermogravimetric analysis of samples with a mass of approximately 10 mg, was performed on a TG209 F1 Iris (Netzsch GmbH & Co. KG, Selb, Germany). The samples were heated up from 27 °C up to 700 °C under argon atmosphere with a heating rate of 10 K/min. For TGA free polydopamine particles were collected out of 100 mL reaction solution after 48 h by filtering off over a 0.2 µm PTFE-filter (Sartorius AG, Göttingen, Germany), washing with 200 mL pure water and drying for 12 h at 60 °C under reduced pressure. Additionally, TGA was performed on a pristine PES membrane sample and PDA-coated PES membranes with different PDA coating times.

### 3.4. Gravimetric Analysis

The mass of the PDA-coating was measured gravimetrically on PES membrane samples (approx. 2 cm × 4 cm), by comparison before and after coating. All samples were dried at 60 °C under reduced pressure and the weight was determined on a lab scale balance R200D (Sartorius AG, Göttingen, Germany), installed in a room free of vibration. After coating the samples were washed and dried as described previously and the mass was determined again.

### 3.5. Water Permeance Measurement (Dead-End)

**Figure 11:**
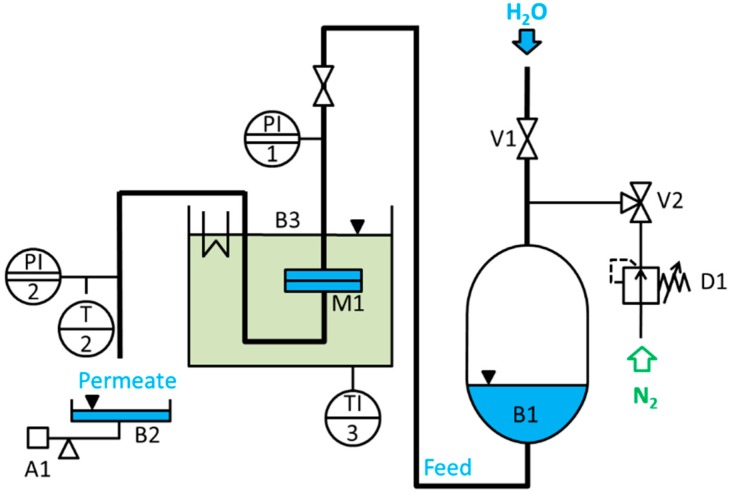
Permeance measurement device (dead-end mode).

Water permeance measurements were performed in dead-end mode using a home-made automatic testing device as shown in Figure 11 at transmembrane pressures of 1.0 bar. The volume was measured gravimetrically every minute by weight acquisition with a Kern EG4200-2NM precision balance with 0.01 g accuracy. In our case all data including pressure, temperature and weight were transmitted to a computer. The effective membrane area was 1.77 cm^2^. These studies were conducted employing demineralized water with an electrical conductivity of ≈0.055 μS·cm^−1^. The pressure normalized permeance (P) was calculated by normalizing the flux by the transmembrane pressure.

(1) $$P=\frac{\Delta V}{A\;\Delta t\;\Delta p}$$

where Δ*V* is the volume of water collected between two mass measurements, *A* is the membrane surface area, Δ*t* is the time between two mass measurements, and Δ*p* is the transmembrane pressure.

Temperature-dependent water permeance measurements were carried out between 3 °C and 50 °C at a transmembrane pressure of 1 bar. The membrane cell (*M1*) is located in a bath, temperature regulated by a F32-ME thermostat of JULABO GmbH (*B3*). Therefore, the temperature is kept at 3 °C for 5 min followed by a linear heating process up to 50 °C within 3 h. For 5 min the temperature stays at 50 °C before cooling down linearly within 3 h to 3 °C again.

The permeance of a membrane can be normalized to 25 °C with the following equation by Crittenden et al. [37]:

(2) $$J_{s}=J_{M}\;x^{T_{s}-T_{m}}$$

where *J_s_* is the normalized permeance at 25 °C, *J_m_* is the measured permeance, *x* is the correction factor, *T_s_* is the standard temperature (in general 25 °C) and *T_m_* is the measured temperature. This normalization can be used between 1 °C and 28 °C and will be important to get very accurate and comparable results since the viscosity of water and properties of the membrane itself are temperature dependent. The correction factor *x* is set to 1.03 for UF membranes, when it was not determined. This factor should be confirmed and adjusted from temperature-dependent water flux measurements [37,43].

### 3.6. Scanning Electron Microscopy

The morphologies of the membrane samples were investigated by scanning electron microscopy (SEM) on a Merlin (Carl ZEISS, Oberkochen, Germany) at a voltage of 0.5 kV. The samples surfaces were sputter-coated shortly for 3 s with a platinum layer (Figure 4A,B).

### 3.7. Atomic Force Microscopy

The surfaces of the membranes were imaged with a Bruker MultiMode 8 (Bruker, Karlsruhe, Germany). The measurements were performed in tapping mode with a TAP150 tip.

### 3.8. Contact-Angle Measurement

Dynamic contact angles were measured on a Drop Shape Analysis System DSA 100 (KRUESS, Hamburg, Germany) with 10 µL water droplets at 24 °C. Contact-Angles were measured 10 s after placing the droplet on the membrane surface.

### 3.9. Stability Test of Polydopamine Coated Membranes against Chemical Cleaning (DIVOS130 and Citric Acid)

**Figure 12:**
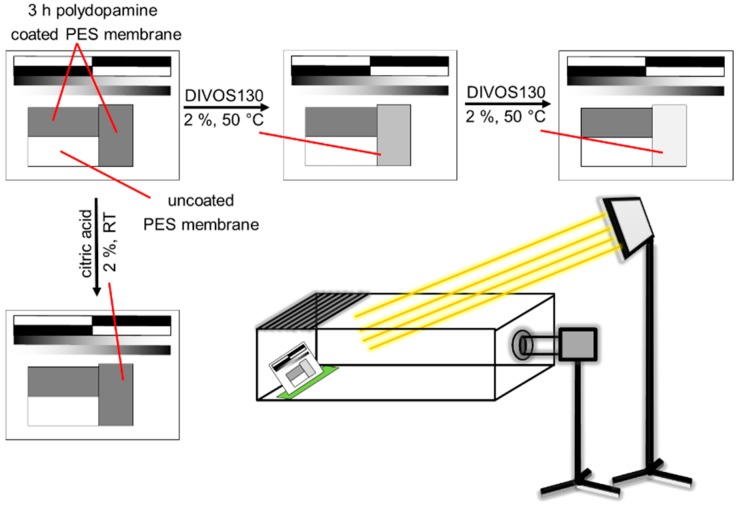
Schematic illustration of the stability test of polydopamine coated membranes against chemical cleaning with DIVOS130 and citric acid. The picture (bottom right) describes the general experimental setup. To keep all distances, angles, light conditions constant the sample was put in a box, open on top with a fixed sample on a sample holder on the left side. Camera settings were fixed in manual mode.

In order to study the stability of polydopamine coated PES membranes, the membranes were treated with a cleaning solution for a certain time (1 min) and the membrane’s colour change by time in grey scale was analysed as a function of time as depicted in Figure 12. The membrane samples (2 × 4 cm) were coated with polydopamine for 3 h. An image illustrating two coated and one uncoated membrane was taken. One of the coated membranes was treated with the cleaning solution, 75 mL of 2% DIVOS130 or 2% citric acid, dried again for 4 min at 60 °C under reduced pressure, placed again to the other samples and a new image was taken. The change of the grey scale value is analysed between 0 and 255 (black to white) whereas the uncoated membrane is defined as white. The reading of the colour value can then be done with any photo software. To exclude fluctuating conditions on photo imaging, the samples were always put at the same position and angle into a self-build box using a fixed sample holder. The box was open on top and a halogen lamp (R75 from Hugo Brennstuhl GmbH, Tübingen, Germany) was used and mounted on a tripod to ensure constant light conditions. Other light sources were switched off. The camera (Nikon D5100 with Nikkor 18-105 objective, Tokyo, Japan) was set to manual mode (f-number F/8, shutter time 1/60 s, ISO-100 and focal lengths 105) to prevent any automatic changes of the photo by the camera and it was also mounted on a tripod at a fixed position.

### 3.10. Fouling Test in Cross Flow Condition

**Figure 13:**
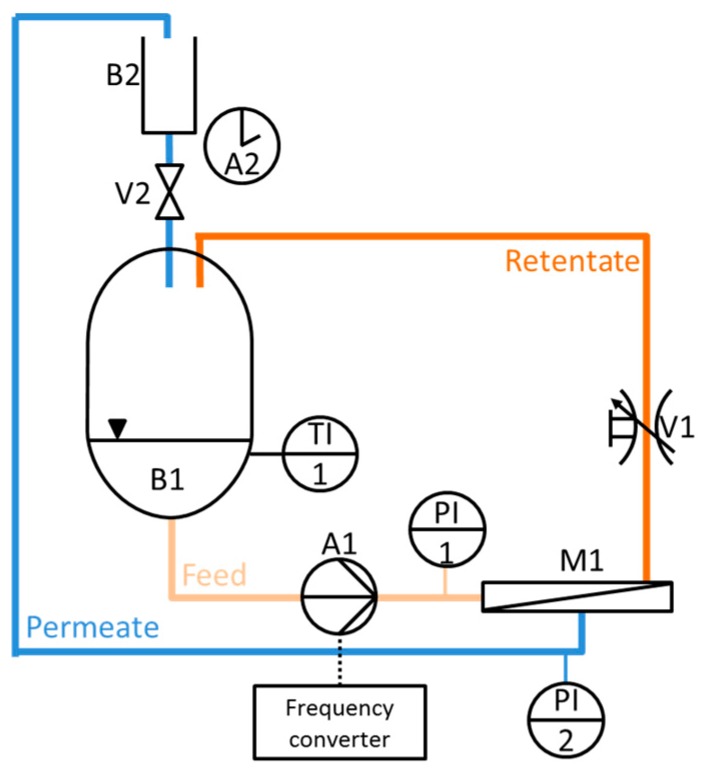
Cross-flow simulation permeance measurement device.

The permeance measurement simulating cross-flow condition were carried out in a specifically designed cross-flow filtration device as schematically depicted in Figure 13. The device consists of a feed tank (B1) with a maximum volume of 4 L, a gear pump (A1) creating a constant flow rate by an adjustable frequency (frequency converter), a regulating stop valve (V1) to regulate the feed pressure, a flat sheet membrane module (M1). The tank (B1) is refrigerated with water in order to keep a constant temperature. The permeate flux is measured with a dropping funnel (B2) with valve (V2) and a stopwatch (A2) manually. The feed and permeate pressures were read with pressure sensors (PI1, PI2), resolution of 1 mbar, in order to calculate the transmembrane pressure. Temperature was measured constantly in the feed tank (TI1).

Fouling tests for natural organic matter (NOM) under cross-flow condition were carried out as follows: The permeance was measured over 60 min with a BSA-solution (61 mg/L in deionized water) as model NOM solution at a transmembrane pressure of 1 bar. Depending on the size of the permeate flux a volume of 50 mL, 25 mL or 15 mL, was collected continuously, over a period of time of approximately 60 s. After 60 min a backwash cycle was carried out for 1 min at 1 bar and the permeance was measured again with BSA-solution.

The permeance decline was calculated with the following equation:

(3) $$\mathrm{Permeance}\;\mathrm{decline}=\frac{P_{1}}{P_{0}}\times 100\%$$

where *P*_0_ is the permeance at the beginning of the fouling test under cross-flow conditions and *P*_1_ is the permeance after 60 min of BSA filtration.

The reversible fouling ratio was evaluated using

(4) $$F\left(r\right)=\frac{P_{2}-P_{1}}{P_{0}}\times 100\%$$

where *F*(*r*) is the reversible fouling ratio in %, *P*_2_ is the permeance after a backwash cycle of 1 min.

Irreversible fouling ratios *F*(*ir*) were calculated as follows

(5) $$F(\mathrm{ir})=\frac{P_{0}-P_{2}}{P_{0}}\times 100\%$$

## 4. Conclusions

This work comprises the use of polydopamine coatings for polyethersulfone membranes as used in oenology. A temperature correction factor for the membranes was determined in order to accurately compare permeance measurements around 20 °C. Permeances and fouling behaviour of the membranes were studied and the stability of the coating layer was tested under basic and acidic conditions. The results can be summarized as follows:

- A good temperature correction factor for polyethersulfone membrane is *x* = 1.012 using the equation: $J_{s}=J_{M}\;x^{T_{s}-T_{m}}$ as suggested by Crittenden et al. [37].
- Permeances of polyethersulfone membranes increase with the polydopamine coating time, here studied up to 240 min.
- The coating layer was studied under basic and acidic conditions, which was investigated by systematically observing the colour change of the coated membranes over treatment time.
- The polydopamine coating layer is not stable in DIVOS130, a basic chemical cleaning agent as used in oenology.
- The polydopamine coating layer is stable in citric acid, which is a common treatment in membrane industry for cleaning fouled membranes.
- Fouling tests under cross-flow conditions show that less fouling occurs after the polyethersulfone membranes are coated with polydopamine.

Future works should try to stabilize polydopamine against basic cleaners, e.g., due to complexation with metal ions [44], adding periodates to the coating mixture [41], covalent bindings or crosslinking. The cross flow conditions and backwashing should be tested over a longer time scale and also backwash cycles should be carried out with chemical cleaning agents in order to test the applicability for this method.

## Figures and Tables

**Table 1:** Gravimetric analysis of polydopamine deposited on PES membranes used in this work.

Membrane	Coating Time (min)	Mass Polydopamine (mg·m^−2^)
Polyethersulfone	0	0 (±25)
Polyethersulfone	60	37 (±25)
Polyethersulfone	120	237 (±25)
Polyethersulfone	180	287 (±25)
Polyethersulfone	240	337 (±25)
Polyethersulfone	1500	2375 (±25)

**Table 2:** Permeance increase of polyethersulfone membranes after polydopamine coating.

Membrane	Coating Time (min)	Average Permeance (L·h^−1^·m^−2^·bar^−1^)	Permeance Increase (%)
Polyethersulfone	0	5191	---
Polyethersulfone	60	5431	4.6
Polyethersulfone	120	5535	6.6
Polyethersulfone	180	5669	9.2
Polyethersulfone	240	5729	10.2
Polyethersulfone	1500	5214	0.4

**Table 3:** Fouling behaviour of pure and polydopamine-modified membranes as calculated flux recovery and fouling ratios.

Membrane	Coating Time (min)	Permeance Decline within 60 min (%)	Reversible Fouling Ratio (%)	Irreversible Fouling Ratio (%)
Polyethersulfone	0	78	48	30
Polyethersulfone	240	55	37	17
